# ForMAX – a beamline for multiscale and multimodal structural characterization of hierarchical materials

**DOI:** 10.1107/S1600577524001048

**Published:** 2024-02-22

**Authors:** K. Nygård, S. A. McDonald, J. B. González, V. Haghighat, C. Appel, E. Larsson, R. Ghanbari, M. Viljanen, J. Silva, S. Malki, Y. Li, V. Silva, C. Weninger, F. Engelmann, T. Jeppsson, G. Felcsuti, T. Rosén, K. Gordeyeva, L. D. Söderberg, H. Dierks, Y. Zhang, Z. Yao, R. Yang, E. M. Asimakopoulou, J. K. Rogalinski, J. Wallentin, P. Villanueva-Perez, R. Krüger, T. Dreier, M. Bech, M. Liebi, M. Bek, R. Kádár, A. E. Terry, H. Tarawneh, P. Ilinski, J. Malmqvist, Y. Cerenius

**Affiliations:** a MAX IV Laboratory, Lund University, Lund, Sweden; b Paul Scherrer Institut, Villigen PSI, Switzerland; c Division of Solid Mechanics, Lund University, Lund, Sweden; d Department of Industrial and Materials Science, Chalmers University of Technology, Gothenburg, Sweden; e Department of Fibre and Polymer Technology, Royal Institute of Technology, Stockholm, Sweden; f Wallenberg Wood Science Center (WWSC), Royal Institute of Technology, Stockholm, Sweden; g Synchrotron Radiation Research, Lund University, Lund, Sweden; h Medical Radiation Physics, Lund University, Lund, Sweden; i Excillum AB, Kista, Sweden; j Institute of Materials, École Polytechnique Fédérale de Lausanne (EPFL), Lausanne, Switzerland; k Department of Physics, Chalmers University of Technology, Gothenburg, Sweden; l FibRe-Centre for Lignocellulose-based Thermoplastics, Department of Chemistry and Chemical Engineering, Chalmers University of Technology, Gothenburg, Sweden; m Wallenberg Wood Science Center (WWSC), Chalmers University of Technology, Gothenburg, Sweden; University of Essex, United Kingdom

**Keywords:** multiscale structural characterization, multimodal structural characterization, hierarchical materials, fibrous materials, small-angle X-ray scattering, wide-angle X-ray scattering, full-field X-ray microtomography

## Abstract

The ForMAX beamline at the MAX IV Laboratory provides multiscale and multimodal structural characterization of hierarchical materials in the nanometre to millimetre range by combining small- and wide-angle X-ray scattering with full-field microtomography. The modular design of the beamline is optimized for easy switching between different experimental modalities. The beamline has a special focus on the development of novel fibrous materials from forest resources, but it is also well suited for studies within, for example, food science and biomedical research.

## Abstract 2

ForMAX is a new beamline at the MAX IV Laboratory, providing multiscale and multimodal structural characterization by combining small- and wide-angle X-ray scattering with full-field tomographic imaging.

## 1. Introduction

Many natural and synthetic materials are hierarchical, exhibiting important structure at several different length scales that govern the material’s properties (Lakes, 1993 ▸; Fratzl & Weinkamer, 2007 ▸; Gibson, 2012 ▸). Wood is an archetypical example, with the assembly of the load-bearing cellulose at nano-, micro- and macroscopic scales determining its mechanical properties. In order to understand the structure–function relationship in such materials, we need access to multiscale structural characterization. Moreover, we need sufficient temporal resolution to allow monitoring of how the structure evolves *in situ* or *in operando* during external stimuli or processing of the material.

The ForMAX beamline of MAX IV addresses this need for structural characterization of hierarchical materials. A key feature is its modular design that allows temporally resolved multiscale structural characterization of bulk materials owing to easy and fast switching between complementary experimental modalities: small- and wide-angle X-ray scattering (SWAXS) in the nanometre regime (Glatter & Kratky, 1982 ▸; Pauw, 2013 ▸) and full-field synchrotron X-ray microtomography (SRµCT) in the micrometre to millimetre regime (Maire & Withers, 2014 ▸). Both of these techniques are applicable to a wide range of materials and suitable for temporally resolved experiments. We foresee that SWAXS will often be carried out in scanning imaging mode using a focused X-ray beam, either as SWAXS-based microscopy (Lichtenegger *et al.*, 1999 ▸; Bunk *et al.*, 2009 ▸), tomography (Feldkamp *et al.*, 2009 ▸; Jensen *et al.*, 2011 ▸) or tensor tomography (Liebi *et al.*, 2015 ▸; Schaff *et al.*, 2015 ▸), covering seven orders of magnitude in length scales and hence being particularly useful for structural characterization of hierarchical materials.

ForMAX is externally funded, with the objective of supporting research on new materials from renewable forest resources. Its construction was funded by the Knut and Alice Wallenberg foundation (https://kaw.wallenberg.org/), while the operation costs for ten years are covered by Swedish industry via Treesearch (https://www.treesearch.se), a national research platform for the development of new materials and speciality chemicals from the forest. Access for both Treesearch members and general users is granted through common calls for proposals, with half of the user beam time reserved for academic and industrial members of Treesearch. For a brief background to ForMAX, see McEntee (2023 ▸).

In the following we outline the technical design of the beamline, and the data acquisition and data processing arrangements, with a focus on the needs of the user. We conclude by providing an initial benchmarking of the beamline and a few examples of multiscale and multimodal structural characterization available on ForMAX.

## 2. Technical design

The combination of SWAXS and SRµCT provides a number of technical challenges, in particular when applied *in situ* or *in operando* to fibrous materials such as wood-based materials:

(i) In SRµCT one monitors the attenuated beam directly downstream of the sample (*i.e.* in the forward scattering direction), while in SAXS one collects scattering data further downstream at small angles. Since the SRµCT full-field microscope blocks the view of the SAXS detector, we have devised a strategy for easy movement of the former in and out of the X-ray beam.

(ii) WAXS from fibrous materials exhibits anisotropy, reflecting the orientation of the crystalline fibers, fibrils or filaments. When mapping out nanoscale orientation in such materials, one needs to be able to collect WAXS data in all directions of the scattering plane (Lichtenegger *et al.*, 1999 ▸). In order to facilitate scanning SWAXS imaging experiments on these materials, we have therefore chosen a custom WAXS detector with a hole in the center, that passes the SAXS signal while simultaneously catching anisotropic WAXS data.

(iii) SWAXS is often carried out at moderate X-ray energies *E* ≃ 10 keV in order to reach small scattering vector moduli *q*, while it is advantageous to carry out SRµCT at higher X-ray energies (≥20 keV) for enhanced phase contrast. As a compromise we operate ForMAX in the 8–25 keV energy range, which is particularly suitable for soft materials.

(iv) Whereas the small divergence of the X-ray beam at the MAX IV 3 GeV storage ring (Tavares *et al.*, 2018 ▸) is beneficial for SWAXS experiments, it limits the natural beam size at the sample position in full-field imaging. As a compromise, we have placed the sample relatively far downstream of the source (42 m from source), while still allowing a reasonable sample-to-detector distance for SAXS experiments. We will install secondary beam-expanding optics in the experimental station to facilitate full-field imaging.

(v) In order to obtain a clean X-ray beam for SAXS experiments, we need to reject higher harmonics of the monochromator by passing the beam via X-ray mirrors. In the full-field imaging mode, the slope errors of the mirrors cause parasitic striation of the X-ray beam. We mitigate the effect of striation by shape compensation of the mirrors.

(vi) Due to the high photon density at fourth-generation sources like the MAX IV 3 GeV storage ring (Tavares *et al.*, 2018 ▸), radiation damage in organic samples is a major issue that we need to assess and mitigate case by case. This also holds true for full-field imaging, which has traditionally been less prone to beam-induced radiation damage due to a large beam size.

(vii) Finally, in order to accomodate various sample environments, such as a rheometer or a mechanical load device with a controlled atmosphere, we need an experimental table that is spacious and has a relatively large load capacity.

**Figure 1:**
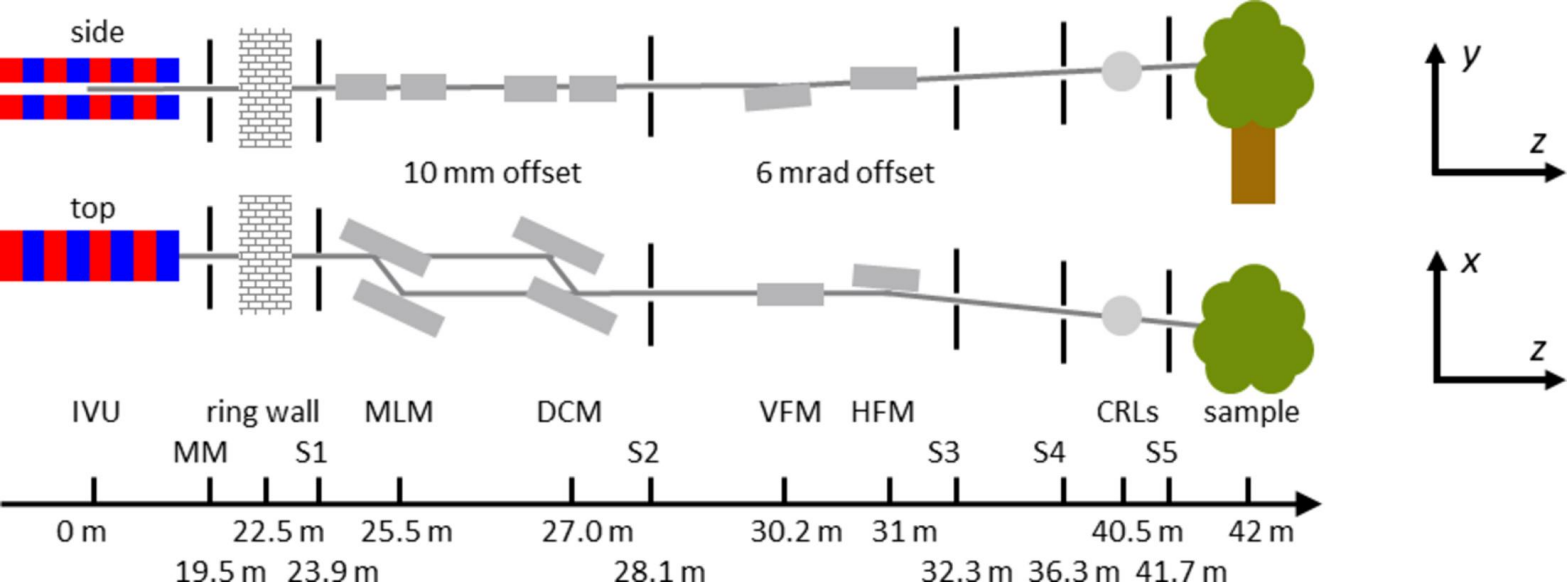
Schematic side and top views of the beamline optics and beam-conditioning components along the beamline (not to scale), with approximate distances from the source at the bottom. Diagnostic components have been omitted for clarity. The MAX IV coordinate system is depicted to the right. Abbreviations are as follows: IVU in-vacuum undulator, MLM multilayer monochromator, DCM double-crystal monochromator, VFM vertically focusing mirror, HFM horizontally focusing mirror, CRLs compound refractive lenses, MM front-end movable mask, S1 white-beam slits and S2–S5 monochromatic slits.

In Table 1 ▸ we summarize the main parameters of the MAX IV 3 GeV storage ring, while in Table 2 ▸ and Fig. 1 ▸ we present the main components of the ForMAX beamline.

Throughout this article, we employ MAX IV’s coordinate system: the lateral *x* axis with positive direction outbound from the ring, the vertical *y* axis with positive direction upwards and the longitudinal *z* axis with positive direction downstream from the source. The positive direction of each rotation around the Cartesian axes (*Rx*, *Ry* and *Rz*) is given by the right-hand rule.

### 2.1. Undulator and front end

**Figure 2:**
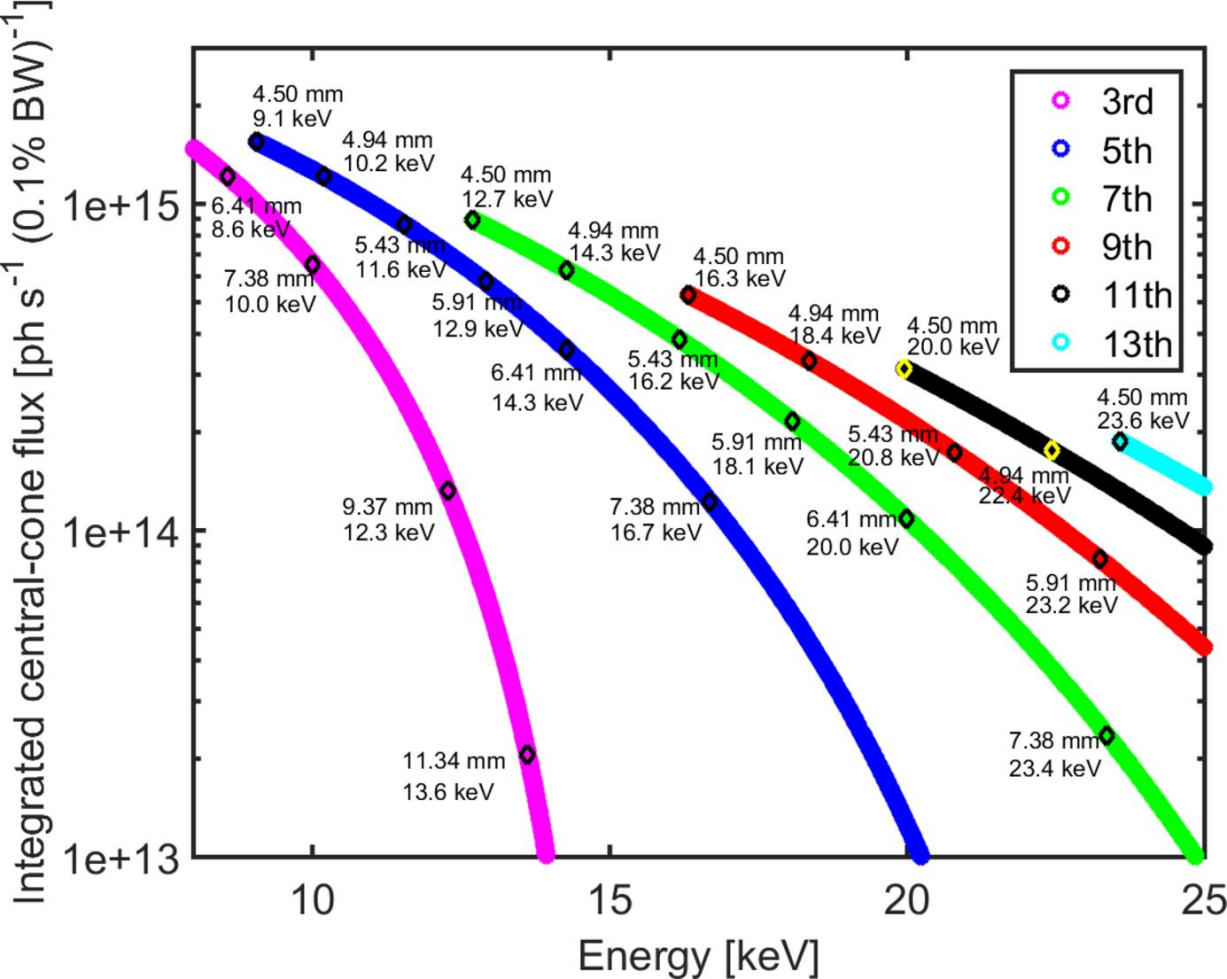
Approximate integrated central-cone flux versus X-ray energy (Kim, 2009 ▸), shown for odd undulator harmonics. Selected undulator gaps are specified for convenience.

ForMAX is equipped with a 3 m long room-temperature in-vacuum undulator from Hitachi Metals. The maximum effective deflection parameter is *K* = 1.89 at the minimum magnetic gap of 4.5 mm and the measured phase error is within specification for all operational gaps. In order to cover the energy range of 8–25 keV, we make use of the fifth to thirteenth harmonics of the undulator as shown in Fig. 2 ▸. Similar to other beamlines around the MAX IV 3 GeV storage ring (Ursby *et al.*, 2020 ▸; Johansson *et al.*, 2021 ▸), the undulator exhibits narrow harmonic peaks, Δ*E* < 100 eV (full width at half-maximum, FWHM). We summarize the main parameters of the undulator in Table 3 ▸.

The front end serves as the interface between the MAX IV 3 GeV storage ring and the ForMAX beamline and was provided by Toyama. It is part of the personal and machine safety systems; it ensures safe access to the optical hutch and safe equipment operation. It includes safety and photon shutters, several fixed and movable masks, various diagnostics components including beam viewers, X-ray beam position monitors, thermocouples and vacuum gauges, as well as vacuum valves to separate different vacuum sections and to safeguard the vacuum of the storage ring in case of vacuum loss in the beamline. The fixed masks remove a vast portion of the undulator radiation power, with the front end typically passing ∼130 W of radiation to the optics hutch at the projected 500 mA ring current. The movable mask, based on two L-shaped GLIDCOP slits with tantalum edges and located ∼19.5 m downstream of the source, is used to define the angular acceptance of the photon beam for the ForMAX beamline.

### 2.2. Primary optics

ForMAX’s primary optics consist of a double-crystal monochromator provided by FMB Oxford, a double-multilayer monochromator by Axilon, dynamically bendable vertical and horizontal focusing mirrors in Kirkpatrick–Baez geometry by IRELEC, a photon shutter by Axilon, and four diagnostics modules by FMB Oxford that host a fixed mask limiting the beamline’s acceptance angle to ≤24 µrad × 36 µrad (*x* × *y*), a high-band-pass diamond filter for heat-load management, a white-beam stop, bremsstrahlung collimators, slits, beam viewers and beam intensity monitors. In the following we will briefly discuss the monochromators and mirrors.

#### 2.2.1. Monochromators

Depending on the experimental needs, ForMAX can be operated using either a double-crystal monochromator (DCM) or a double-multilayer monochromator (MLM). In line with several other hard X-ray beamlines at MAX IV (Ursby *et al.*, 2020 ▸; Johansson *et al.*, 2021 ▸; Kahnt *et al.*, 2021 ▸), we have chosen a horizontal deflection geometry for both monochromators to maximize their stability. Owing to the relatively high X-ray energy on ForMAX, the energy-dependent polarization factors are >0.75 and >0.99 in the full energy range for the DCM and MLM, respectively. In order to facilitate switching between monochromators, both employ the same fixed-exit design with 10 mm inboard offset.

The horizontally deflecting Si(111) DCM is positioned 27 m from the source. We note that the small horizontal offset between the crystals allows for a compact and rigid design with excellent stability, as shown elsewhere (Kristiansen *et al.*, 2016 ▸). In ForMAX’s case, the 50 mm long upstream crystal is mounted directly on the Bragg goniometer (*Ry*) without any other motorized axes, while the 100 mm long downstream crystal has additional motorized adjustments for pitch *Ry*, roll *Rz* and perpendicular motion. The monochromator is equipped with motorized lateral *x* and vertical *y* translations. Both crystals are side cooled by clamping them to liquid-nitrogen-cooled Cu blocks; the high heat load of the upstream crystal requires direct cooling of the Cu block, while indirect cooling of the Cu block by braids is sufficient to manage the lower heat load of the downstream crystal.

The horizontally deflecting MLM, in turn, is positioned 25 m from the source. While it is foreseen to be used almost exclusively for full-field imaging experiments requiring high temporal resolution, it may also find use in niche photon-hungry scattering experiments. Both multilayer mirrors consist of flat 180 mm long Si(100) substrates covered with separate stripes of 200 layers of W/B_4_C and 250 layers of Ru/B_4_C. Each multilayer stripe has a period of ∼2.4 nm and ∼1.6 nm B_4_C layer thickness, optimized for the energy range of the beamline. The bandpass of the MLM, Δ*E*/*E* ≃ 1% by design, is larger than the width of an individual harmonic peak of the undulator. The Bragg rotation *Ry* of the monochromator, fine roll *Rz* of the upstream mirror and fine pitch *Ry* of the downstream mirror are all realized by linear actuators and special flexure arrangements. Due to the large angular range of the monochromator, a longitudinal *z* translation of the downstream multilayer assembly is needed. The motorized motions include the lateral *x* and vertical *y* translations of the monochromator as well as the perpendicular translation of the downstream multilayer assembly. Due to the significantly smaller Bragg angle compared with the DCM, and hence a larger X-ray beam footprint, it suffices to cool both multilayer mirrors indirectly by braids from water-cooled Galinstan baths.

#### 2.2.2. Mirror system

The mirror system consists of vertically (VFM) and horizontally (HFM) focusing (and deflecting) mirrors in Kirkpatrick–Baez geometry, housed inside a single vacuum chamber. Each mirror has a 650 mm optical length and works at a fixed incidence angle of 3 mrad. The mirrors serve two main purposes. First, they provide harmonic rejection. In order to cover the wide energy range of the beamline, each mirror from Insync has three separate stripes of Si, Rh and Pt. Second, each mirror can be independently bent to radii between ∼5 and 100 km, allowing us to focus at the nominal sample position or any position downstream thereof, collimate the beam, or essentially operate without focusing. In practice, the mirror bending is achieved by applying two controlled bending moments (monitored by strain gauges) at the upstream and downstream ends of the mirror in a four-point bending configuration. Each mirror is equipped with a limited number of stiff motorized axes to maximize stability: lateral *x* and vertical *y* granite translation stages, as well as pitch rotation (*Rx* for VFM, *Ry* for HFM) employing a high-resolution actuator and flexure parts. The HFM is also equipped with a similar motorized roll rotation *Rx* by combining a high-resolution actuator and flexure parts.

Mirror slope errors cause striation of the downstream X-ray beam which is a nuisance when operating the beamline in unfocused mode during SRµCT experiments. In order to minimize this effect, each bender is equipped with a set of five spring actuators or so-called shape compensators. The residual slope errors for the flat geometry are ∼0.11 and ∼0.13 µrad for the VFM and HFM, respectively. In the nominal elliptical shape for focusing, the mirrors show residual slope errors ≤0.19 µrad for each stripe.

### 2.3. Experimental station

**Figure 3:**
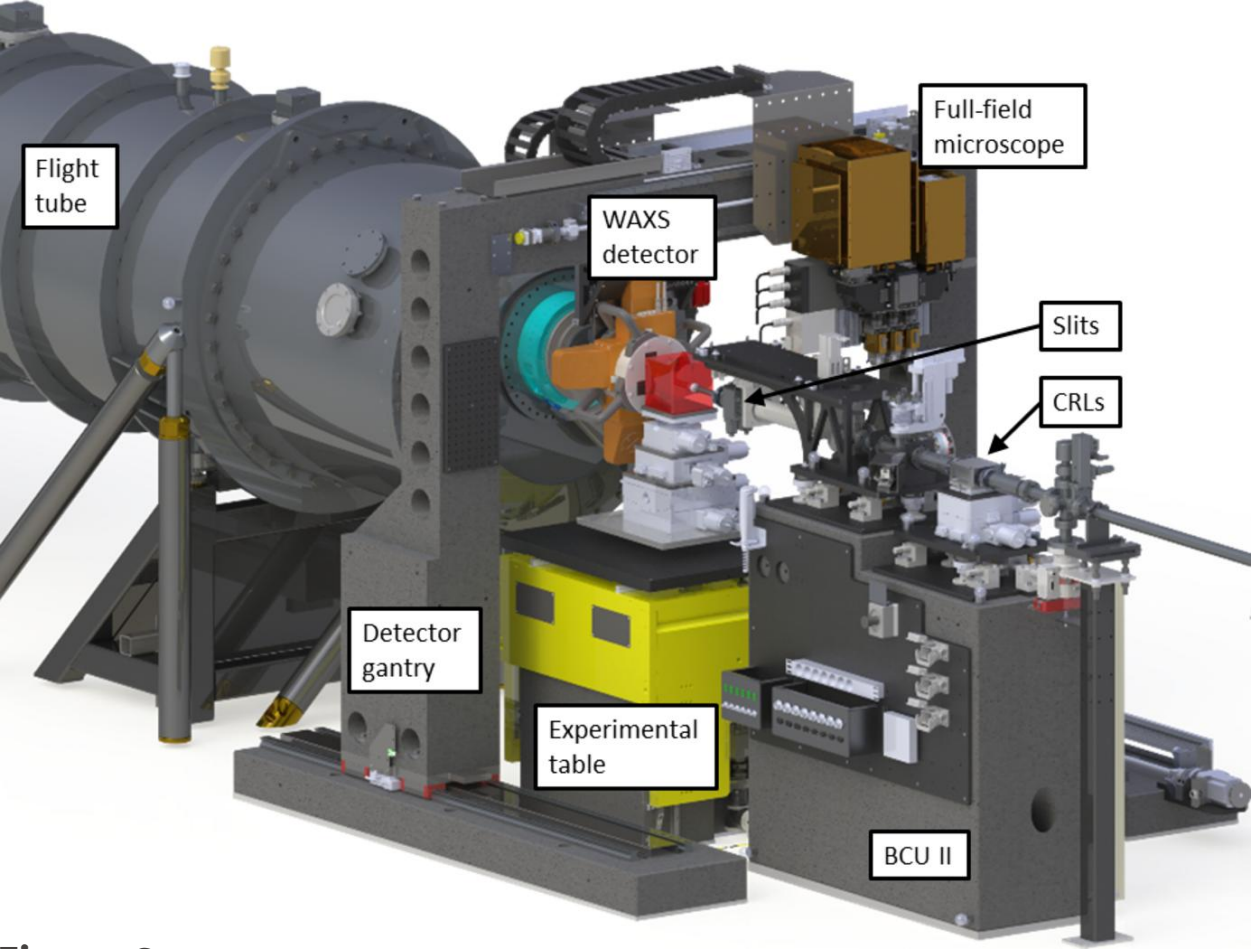
The experimental station of the ForMAX beamline. The main components shown include the downstream BCU II, the experimental table, the detector gantry, with the WAXS detector and full-field microscope mounted onto it, and the evacuated flight tube, hosting the SAXS detector. The CRLs and the monochromatic slits of BCU II are also highlighted for convenience. In the SWAXS setup depicted in the figure, the vacuum nose cone, onto which the WAXS detector is mounted, is connected to the flight tube using a bellow, while the full-field microscope is translated out of the X-ray beam.

The major components of the experimental station shown in Fig. 3 ▸ – two beam-conditioning units (BCUs), an experimental table, a detector gantry and a flight tube – have been custom designed at MAX IV. Due to the different, and sometimes mutually competing, technical requirements of SWAXS and SRµCT as outlined above, we gave special attention to the integration of these components into a single instrument. Because of the modular nature of the experimental station, as described below, we have installed a dedicated programmable logic controller (PLC) system to ensure its safe operation. In order to mitigate the effect of parasitic scattering in the small-angle regime, which hampers SAXS studies of weakly scattering bio-based materials such as low-concentration suspensions of cellulose nanoparticles, all windows in the X-ray beam path of the ForMAX beamline are single crystalline. Finally, we have dedicated space between the BCUs to assemble a setup for X-ray multi-projection imaging (XMPI) (Villanueva-Perez *et al.*, 2018 ▸, 2023 ▸).

#### 2.3.1. Beam-conditioning units

The experimental station hosts two BCUs, positioned approximately 36 and 41 m downstream of the source. The upstream BCU (called BCU I) includes a fast shutter, a pneumatic filter unit and a set of monochromatic slits. In the near future, it will also host an X-ray prism lens that allows beam expansion in the ∼5 mm range for full-field tomographic imaging experiments. The downstream BCU (BCU II) includes a beam viewer, two Si diodes for X-ray beam flux monitoring, a set of monochromatic slits, a set of compound refractive lenses optimized to provide a microfocus X-ray beam at 16.3 keV for scanning SWAXS experiments, and the possibility of mounting a simple off-axis optical microscope for visual monitoring of the sample. In order to minimize the X-ray path in air for different setups, the exit vacuum window of BCU II is motorized along the beam path. Finally, all slits in the experimental station are so-called hybrid scatterless slits (Li *et al.*, 2008 ▸), with single-crystal InP wafers mounted on tungsten carbide blades in order to suppress parasitic X-ray scattering.

#### 2.3.2. Experimental table

The experimental table is located 42 m downstream of the source and is based on a concept developed at the ALBA synchrotron (Colldelram *et al.*, 2010 ▸). The table provides flexibility for sample environment mounting in terms of an available top surface of 800 mm × 800 mm, a load capacity of 200 kg, large lateral and vertical translation ranges of 200 mm each, and up to ∼520 mm space between the top surface and the X-ray beam.

The base of the experimental table is a stable and stiff granite block. For vertical *y* motion of the table (∼0.3 µm resolution^1^) we make use of two (upstream and downstream) motorized steel plates that are driven by ball screws with linear guides and actuated by stepper motors. Flexure hinges on the steel plates allow fine tuning of the pitch *Rx* (20 mrad range, ∼0.4 µrad resolution). We have added the lateral *x* motion (∼0.3 µm resolution) on top of the assembly, again driven by a ball screw with linear guides.

#### 2.3.3. Detector gantry

The granite detector gantry, located by the experimental table, hosts the WAXS detector and the full-field microscope for SRµCT. It has five independent motions:

(i) The longitudinal motion of the gantry along the X-ray beam path (∼1500 mm range, 10 µm resolution),

(ii) Lateral (∼700 mm) and vertical (∼20 mm) motions of the WAXS detector (10 µm resolution each), and

(iii) Lateral (∼700 mm) and vertical (∼30 mm) motions of the full-field microscope (1 µm resolution each).

We have verified, by measuring the vibrations of the microscope tip with a laser Doppler vibrometer (one minute average, integrated 4–100 Hz), that the amplitudes are <20 nm in both lateral and vertical directions (root-mean-square, r.m.s.).

The above motions permit easy and independent movement of the WAXS detector and the full-field microscope in and out of the X-ray beam, thus providing a number of different experimental modes:

(i) In the SWAXS setup (see Fig. 3 ▸), we center the X-ray beam on the WAXS detector, while the SAXS signal (and unscattered beam) passes through the central hole of the WAXS detector and impinges on the SAXS detector (and the central beam stop). In this setup, we translate the full-field microscope out of the X-ray beam path. In the SAXS setup, in turn, we also translate the WAXS detector out of the X-ray beam path and mount an evacuated nose cone onto the flight tube to minimize the air path downstream of the sample.

(ii) In the SRµCT setup, we translate the WAXS detector out of the path of the X-ray beam. As a safety measure we close a gate valve at the entrance of the flight tube, to avoid X-ray exposure of the SAXS detector.

(iii) In the combined SAXS and SRµCT setup, we align the SAXS detector with the X-ray beam, and translate the full-field microscope vertically in and out of the X-ray beam path for full-field imaging and scattering modes, respectively. The vertical translation of the microscope out of the X-ray beam path takes ∼15 s. A combined SWAXS and SRµCT setup is also possible, but the accessible SAXS and WAXS angular ranges are limited by space restrictions.

#### 2.3.4. Flight tube

In order to minimize (i) absorption of the scattered X-ray beam and (ii) parasitic X-ray scattering from air, we have placed the SAXS detector on a motorized detector trolley inside a 9 m long and 1 m diameter evacuated vacuum vessel operating at ∼10^−3^ mbar. We have also mounted a motorized central beam stop, made from tungsten and equipped with a GaAs diode for monitoring the flux of the transmitted X-ray beam, on the detector trolley. The motorized longitudinal motion of the detector trolley permits easy switching of the nominal sample-to-detector distance in the range of ∼800–7600 mm,^2^ while the independent motorized lateral and vertical motions allow users to position the SAXS detector freely with respect to the direct X-ray beam. We have mechanically decoupled the rail system of the detector trolley from the vacuum vessel, thereby isolating the trolley motion from vibrations and vacuum-induced deformations of the vessel.

### 2.4. Sample manipulation

**Figure 4:**
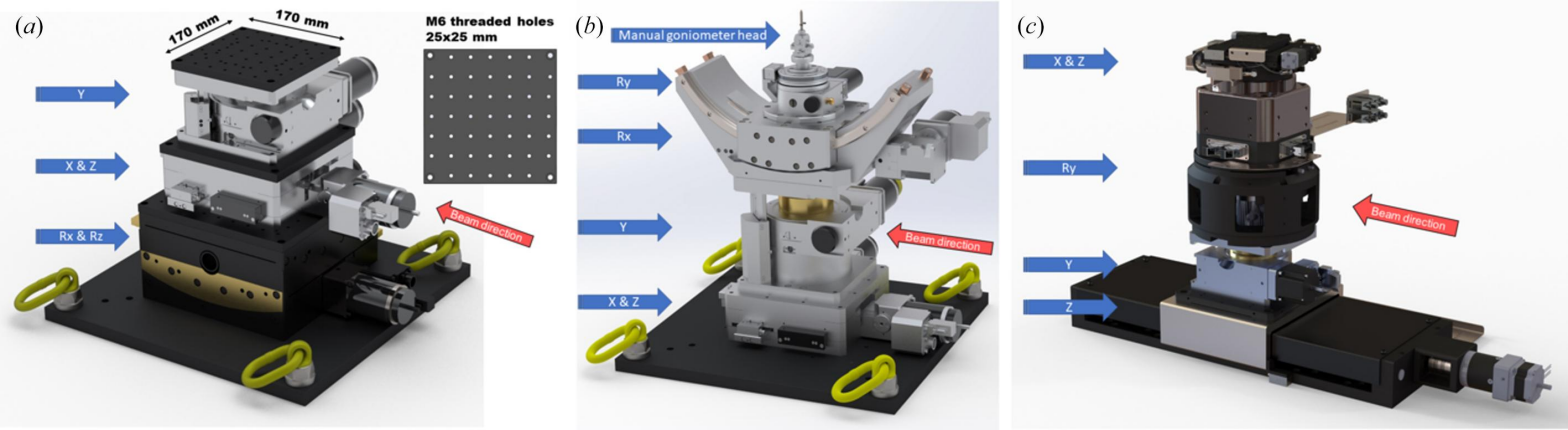
Assembly of stages for sample manipulation in (*a*) SWAXS, (*b*) scanning SWAXS and (*c*) SRµCT experiments.

ForMAX offers a number of experimental techniques, each with specific requirements with respect to sample manipulation. In order to meet different user needs, ForMAX is equipped with three separate stacks of stages for sample manipulation:

(i) For SWAXS experiments, we provide a high-load (≤1500 N) five-axis assembly from Huber as shown in Fig. 4 ▸(*a*). It consists, from bottom to top, of motorized pitch *Rx* (±13°), roll *Rz* (±12°), lateral *x* (±25 mm), longitudinal *z* (±25 mm) and vertical *y* (±20 mm) axes. In order to simplify mounting of sample holders or environments, we have added an optical breadboard with a 25 mm × 25 mm grid of centered ISO metric M6 threaded holes on top of the stages. The nominal distance between the top surface and the center of rotation is 49 ± 20 mm, but this can be increased owing to the modular nature of the assembly of stages.

(ii) For scanning SWAXS experiments, we provide another assembly with five degrees of freedom by Huber [Fig. 4 ▸(*b*)]. The base consists of motorized lateral *x* (±25 mm), vertical *y* (±10 or ±45 mm, depending on resolution and speed requirements) and longitudinal *z* (±25 mm) axes for 2D scanning and adjustment of the sample along the X-ray beam path. On top of these we have mounted a yaw *Ry* axis which, combined with the translation stages below, allows SWAXS tomographic imaging. Finally, we have added a large-range custom pitch axis *Rx* (±45°) for SWAXS tensor tomography experiments. A manual five-axis goniometer head (Huber, model 1002 or 1005) on top of the assembly enables fine alignment of the sample.

(iii) In SRµCT experiments we employ a five-axis assembly from Lab Motion as shown in Fig. 4 ▸(*c*). It consists, from bottom to top, of a motorized longitudinal *z* axis (∼380 mm range) for propagation-based phase-contrast imaging, a vertical *y* axis (±20 mm) for helical imaging, an air-bearing tomographic yaw axis *Ry*, coupled with a rotary union accomodating a fluid slip ring, and horizontal *xz* axes (±5 mm each) for sample alignment. The electrical slip ring is equipped with 15 spare wires for integration of sample environments. The maximum rotation speed of the yaw stage is 720 revolutions per minute, allowing SRµCT experiments with temporal resolution up to ∼20 Hz. The assembly is modular and is typically operated without the vertical axis and the rotary union. In order to facilitate mounting of sample holders or environments, we have installed an optical breadboard with a 12.5 mm × 12.5 mm grid of centered ISO metric M6 threaded holes on top of the stages.

We further note that we can combine the air-bearing tomographic rotation stage with the linear scanning SWAXS stages in a modular setup, allowing combined high-resolution SRµCT and 2D/3D scanning SWAXS experiments without the need to re-mount the sample upon changing experimental modality.

## 3. Data acquisition and processing

Data acquisition and processing greatly affect the user experience. In the following, we briefly review how these are managed on the ForMAX beamline.

### 3.1. X-ray detection systems

For SWAXS experiments, ForMAX is equipped with two megapixel hybrid photon-counting detectors that provide high resolution, high dynamic range and low noise. The SAXS detector is a vacuum-compatible Dectris EIGER2 X 4M (Donath *et al.*, 2023 ▸). The WAXS detector, in turn, is a custom X-Spectrum Lambda 3M (Pennicard *et al.*, 2013 ▸). In Table 4 ▸ we provide technical details about both detectors.

The range of scattering vector modulus *q* covered by the SAXS detector depends on the X-ray energy, the sample-to-detector distance (SDD), the positioning of the SAXS detector in the scattering plane and the size of the central beam stop (at the moment 4–5 mm diameter); assuming that the SAXS detector is centered on the direct X-ray beam, the accessible SAXS *q* range varies from *q* ≃ 0.01–0.5 nm^−1^ at minimum X-ray energy and maximum SDD to *q* ≃ 0.25–10 nm^−1^ at maximum energy and minimum SDD.

**Figure 5:**
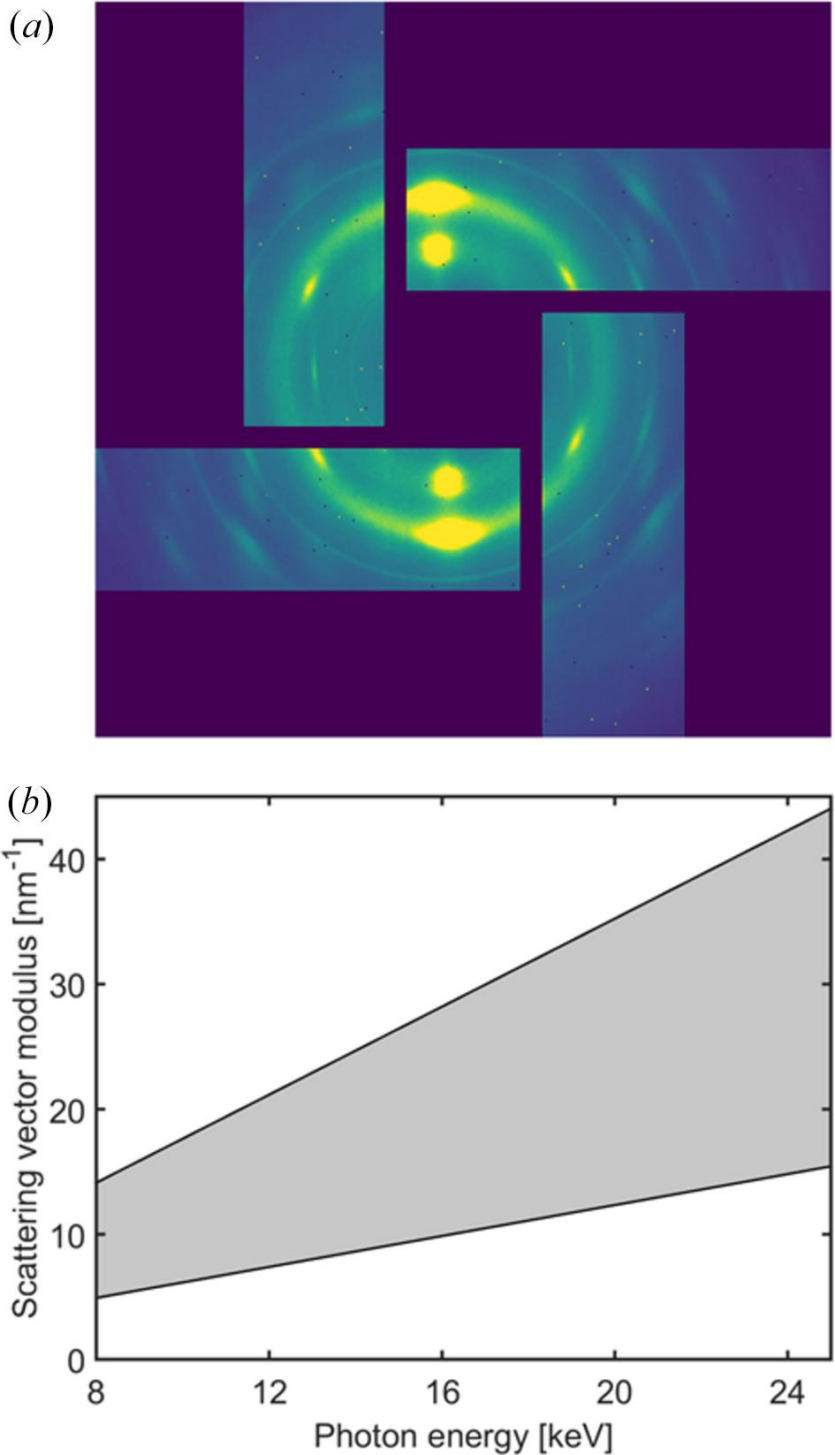
WAXS on ForMAX using the custom Lambda 3M ‘windmill’ detector. Panel (*a*) exemplifies a diffraction pattern from a piece of wood, while panel (*b*) shows the nominal accessible range of scattering vector moduli *q* (gray area) versus X-ray energy.

The custom WAXS detector warrants a more detailed discussion. In order to facilitate scanning SWAXS experiments from fibrous materials, it has a hole in the center to pass the SAXS signal (and the direct X-ray beam), while simultaneously allowing us to collect WAXS data in all directions of the scattering plane [Fig. 5 ▸(*a*)]. It is mounted onto an evacuated nose cone and connected to the flight tube via a bellow, thereby minimizing parasitic air scattering in the SAXS regime. At the nominal SDD of 135 mm, we can collect WAXS data at scattering angles 2θ = 7–20° in all directions of the scattering plane, yielding the energy-dependent range of accessible scattering vector moduli *q* = 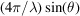 shown in Fig. 5 ▸(*b*). We note that there is an ∼100 mm path of air between the sample and the entrance window of the flight tube when using the WAXS detector, adding to the parasitic background scattering in the SAXS regime. Finally, due to the thickness of the full-field microscope, SDD ≥ 235 mm in the combined SWAXS and SRµCT experiments, essentially halving (i) the energy-dependent minimum and maximum *q* of Fig. 5 ▸(*b*) and (ii) the accessible scattering angles 2θ in the SAXS regime due to shadowing of the flight-tube entrance window, hence in practice limiting these experiments to X-ray energies ≥20 keV.

For SRµCT experiments, ForMAX is equipped with a high-resolution full-field microtomography detection system encompassing two main components – an optical microscope and an sCMOS camera. The transmitted X-ray beam is converted by a scintillator into visible light, which is in turn magnified by the optical microscope and recorded by the sCMOS camera. The white-beam optical microscope from Optique Peter has motorized triple objective lens and dual camera port configurations for simple switching of magnification and sCMOS camera, respectively. We can operate the microscope with 2×, 5×, 7.5×, 10× and 20× objectives, depending on the required effective pixel size and field of view.

Currently we employ two sCMOS cameras for high-resolution imaging at limited speed, the Hamamatsu ORCA Lightning and the Andor Zyla. We are also equipped with a high-speed Photron FASTCAM Nova sCMOS camera to allow ∼20 Hz SRµCT, *i.e.* the maximum temporal resolution allowed for by the tomographic rotation stage. We summarize the technical details of the sCMOS cameras in Table 5 ▸.

**Figure 6:**
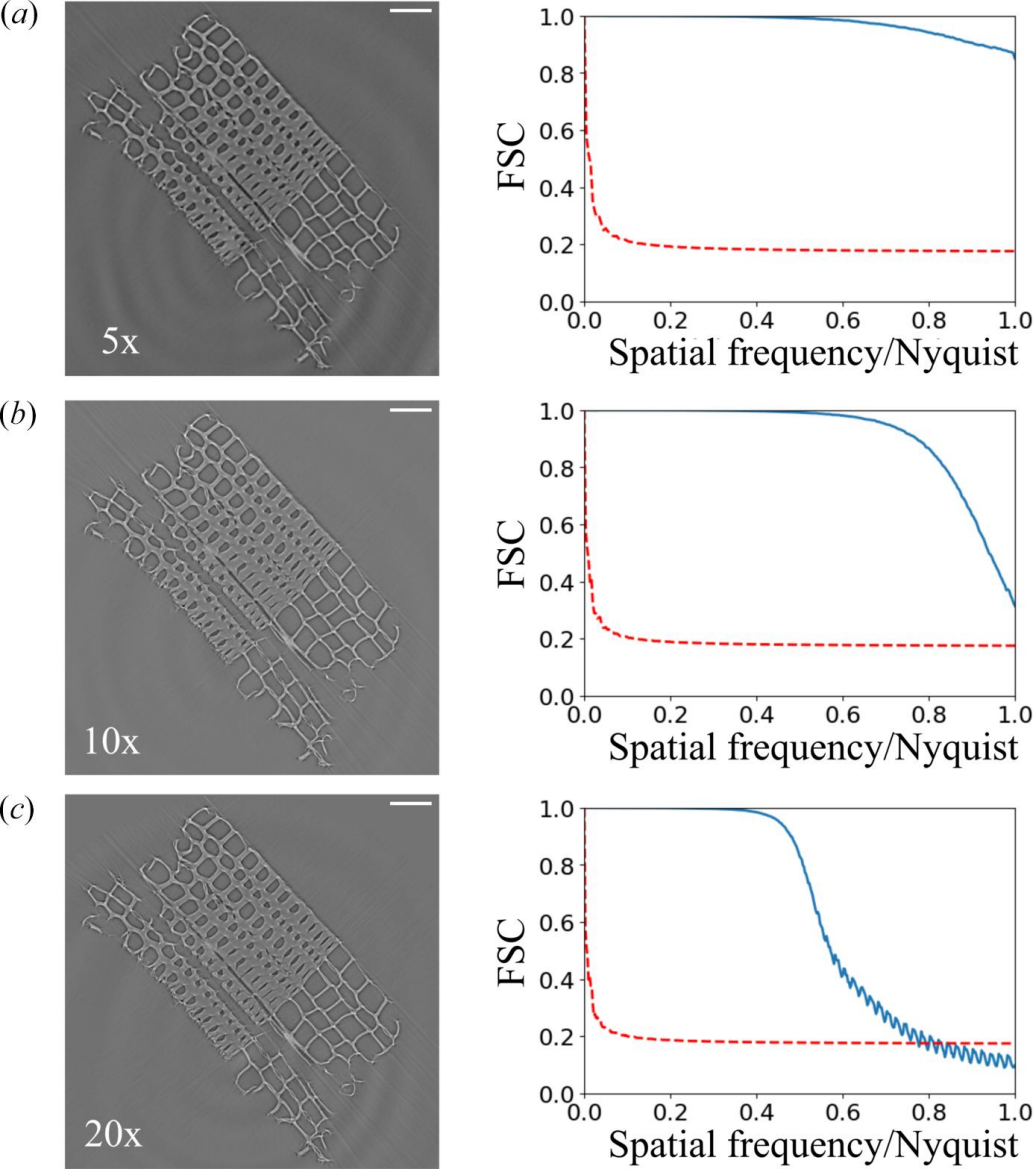
SRµCT resolution evaluation on ForMAX. The left-hand column contains the reconstructed slices for (*a*) 5×, (*b*) 10× and (*c*) 20× magnification (100 µm scale bars). The right-hand column contains the results of the Fourier shell correlation (FSC) versus spatial frequency (normalized by the Nyquist frequency) for each of the magnifications (blue curves) and the half-bit error curve (dashed red curves).

We have evaluated the performance and resolution of the presented SRµCT system. For this evaluation, we used the full-field microscope with 5×, 10× and 20× magnification coupled to the Andor Zyla camera (physical pixel size 6.5 µm), resulting in 1.3, 0.65 and 0.325 µm effective pixel sizes, respectively. The reconstructed slices for the different magnifications of a wood sample are presented in the left-hand column of Fig. 6 ▸. We also evaluated the resolution over the 3D reconstructed volume using the Fourier shell correlation (FSC) together with the half-bit threshold criterion (van Heel, 1987 ▸; van Heel & Schatz, 2005 ▸), as depicted in the right-hand column of Fig. 6 ▸. We observe that the ForMAX instrument retrieves Nyquist-limited resolution for the 5× and 10× magnifications, *i.e.* 2.6 and 1.3 µm resolution, respectively. For the 20× magnification, the retrieved resolution was around 3 pixels, which corresponds to 0.975 µm. Thus, the ForMAX instrument is ideal for characterizing objects in three dimensions with micrometre resolution.

### 3.2. Control system

ForMAX’s control system is based on *Tango* (Chaize *et al.*, 1999 ▸), an open-source control system that is in use at several European synchrotron facilities. On top of *Tango* we employ *Sardana* (Coutinho *et al.*, 2011 ▸), a software environment for *e.g.* controlling motors, acquiring signals and running macros. We have optimized the scan routines for the specific needs of ForMAX, such as reducing the overhead per line in continuous *xy* mesh scans to <1 second for scanning SWAXS applications. From a hardware point of view, the majority of our motorized axes are based on stepper motors controlled by *IcePAP* (Janvier *et al.*, 2013 ▸) and we make use of *PandABoxes* for synchronization of the experiments (Zhang *et al.*, 2017 ▸).

### 3.3. Data pipelines

All detectors are integrated into the beamline control system via dedicated detector servers, utilizing detector-specific software development kits (SDKs) running on detector control units (DCUs) for detector control and data readout. Low-level image processing such as flat-field correction is either applied by default (for hybrid pixel detectors; SWAXS) or in the image reconstruction (for sCMOS cameras; SRµCT), while low-level acquisition parameters such as acquisition time, number of frames and photon-counting threshold energy are accessible to the user. Finally, the data are streamed via high-speed Ethernet connections to MAX IV’s central data storage^3^ and saved together with metadata in the hierarchical data format 5 (HDF5). The data are stored for at least seven years.

**Figure 7:**
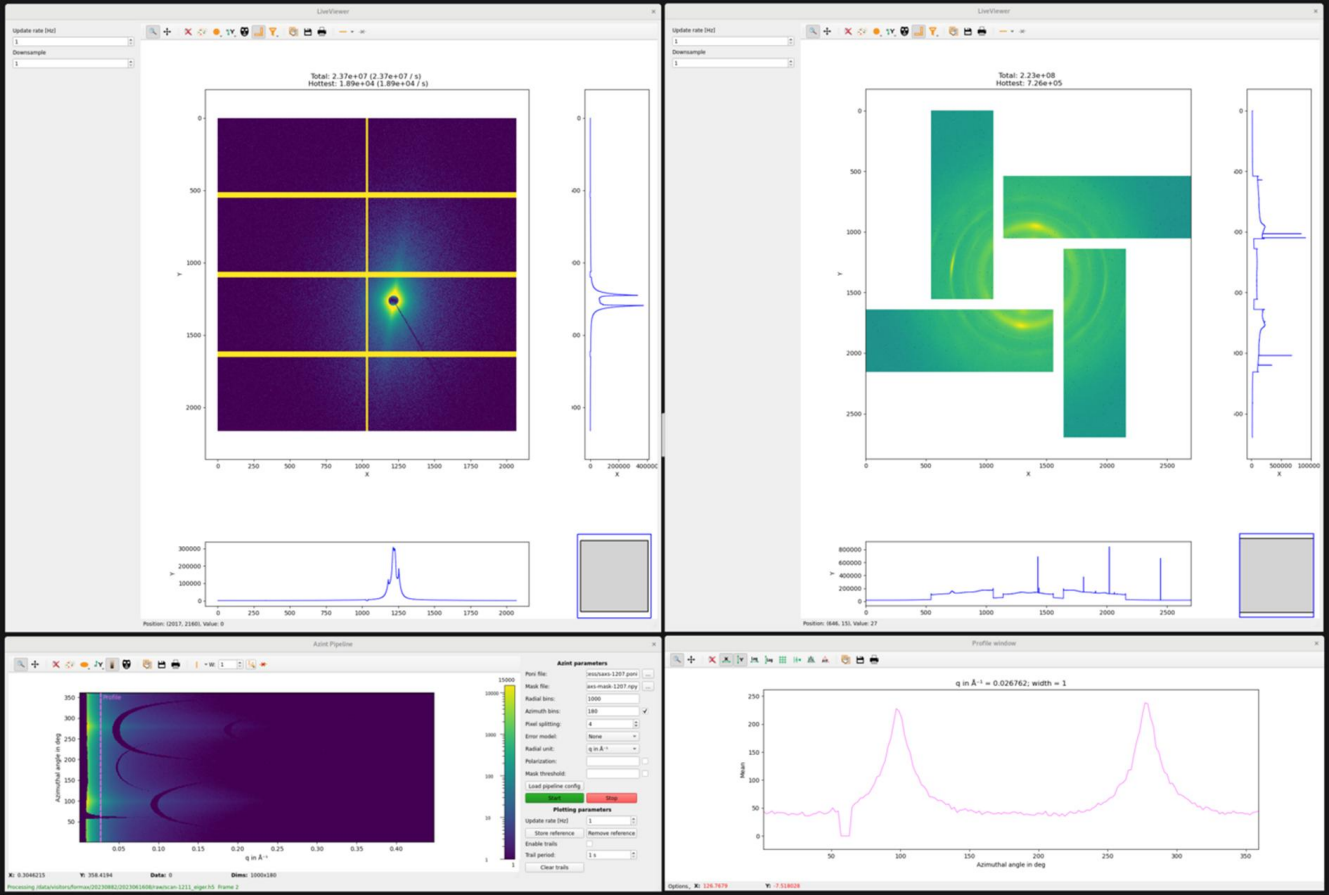
A snapshot from the beamline control computer, exemplifying the live plotting of SWAXS data. The top panel shows as-measured SAXS (left) and WAXS (right) data collected from a wood sample. The bottom left-hand panel illustrates reduced 2D *I*(*q*, φ) data in the SAXS regime. The line profile of the reduced 2D data, shown in the bottom right-hand panel, corresponds to an annular integral of the as-measured SAXS data and is convenient for monitoring anisotropy in the scattering data. Similar live plotting of reduced WAXS data is available on the beamline.

In parallel with data streaming and storage of the as-measured scattering data, our SWAXS data pipeline reduces the data to a more user-friendly format. The data reduction is carried out using the Python implementation of *MatFRAIA* (Jensen *et al.*, 2022 ▸), based on a matrix-multiplication algorithm for radial and azimuthal integration, and is faster than the maximum frame rate of the EIGER detector. We reduce the SWAXS data into both 1D *I*(*q*) and 2D *I*(*q*, φ) formats, where *I* denotes the scattering intensity and φ the azimuthal angle. We emphasize that the fast data reduction into so-called ‘cake plots’, *I*(*q*, φ), is particularly convenient for monitoring anisotropic scattering from fibrous materials in (scanning) SWAXS experiments. For calibration and masking of the detectors we utilize *PyFAI* (Kieffer *et al.*, 2018 ▸), which is well known in the user community. Finally, in order to facilitate monitoring of the experiment, we plot either the radial integral *I*(*q*) or the ‘cake plot’ *I*(*q*, φ) in both SAXS and WAXS regimes in live mode. In Fig. 7 ▸ we present a snapshot from the beamline control computer, exemplifying the live plotting of reduced SWAXS data.

In SRµCT experiments, we take a different approach for the data pipeline. In line with community convention, we collect projections as well as flat- and dark-field images using dedicated scan routines and save all data in common HDF5 files. In order to improve user friendliness further, we are currently in the process of implementing live tomographic reconstructions for SRµCT experiments.

### 3.4. Data analysis and image reconstructions

ForMAX allows a wide range of SWAXS, scanning SWAXS and SRµCT experiments, each with their unique requirements with respect to on-line data analysis. In order to support all these different experiments, we provide up-to-date *Jupyter Notebook* templates for our users. For SRµCT experiments, the script for tomographic reconstruction includes the option to perform phase retrieval for single-distance propagation-based phase-contrast tomography, in addition to standard absorption contrast tomography reconstruction. We plan to implement further developments continuously, including the aforementioned live tomographic reconstructions for SRµCT experiments.

SAXS tensor tomography (SASTT), which combines concepts of scanning SAXS with SRµCT to retrieve not solely scattering intensity measures but the full 3D reciprocal-space map within each voxel of the tomogram, is a special case due to the high demands on image reconstruction. Data acquisition must be matched with sufficient computational resources to allow reconstructions of the 3D reciprocal-space map, ideally already during the experiment, to evaluate the quality of the measurements. On ForMAX, we have implemented *Jupyter Notebook* templates for projection alignment and apply the software package *Mumott* (https://mumott.org/) to perform SASTT reconstructions. In the future, we plan to update these notebooks continuously to remain up to date and match further developments and improvements of the reconstruction algorithm. Details about *Mumott* can be found in a recent publication by Nielsen *et al.* (2023 ▸).

## 4. Benchmarking

In this section we report on initial benchmarking of the main X-ray beam properties on ForMAX.

### 4.1. Beam size

The dynamically bendable mirrors provide a means of varying the lateral and vertical beam size at the sample position over a large range. In the unfocused mode, we obtain an FWHM beam size of ∼0.8 mm × 1.3 mm (*x* × *y*) using the DCM, while the larger bandpass of the MLM yields a beam size of ∼1.3 mm × 1.5 mm. At the other extreme, we can focus the beam down to ∼55–60 µm × 10–15 µm at the sample position using either monochromator. In this case, the lateral beam size is limited by the source size and imaging geometry, while the vertical beam size is limited by the slope errors of the mirrors. The dependence of the beam size on the undulator harmonic is negligible.

In order to decrease the beam size further at the sample position, we have installed compound refractive lenses (CRLs) ∼1.5 m upstream of the nominal sample position. At the moment we employ a stack of 16 radiation-resistant SU-8 polymeric lenses from Microworks, optimized for 16.3 keV X-rays and yielding a FWHM beam size of ∼10 µm × 2 µm at the sample position. This is similar to the beam size typically available on third-generation SWAXS beamlines with microfocus capability (Buffet *et al.*, 2012 ▸; Smith *et al.*, 2021 ▸).

The natural beam size on ForMAX limits SRµCT experiments on large samples. This limitation can be partly overcome by stitching images, but at the expense of temporal resolution. We will soon also install an optional overfocusing SU-8 X-ray prism lens from Microworks ∼5.4 m upstream of the nominal sample position, yielding an energy-dependent X-ray beam size of ∼5 mm × 5 mm or larger at the sample position in combination with the DCM.

### 4.2. Flux

**Figure 8:**
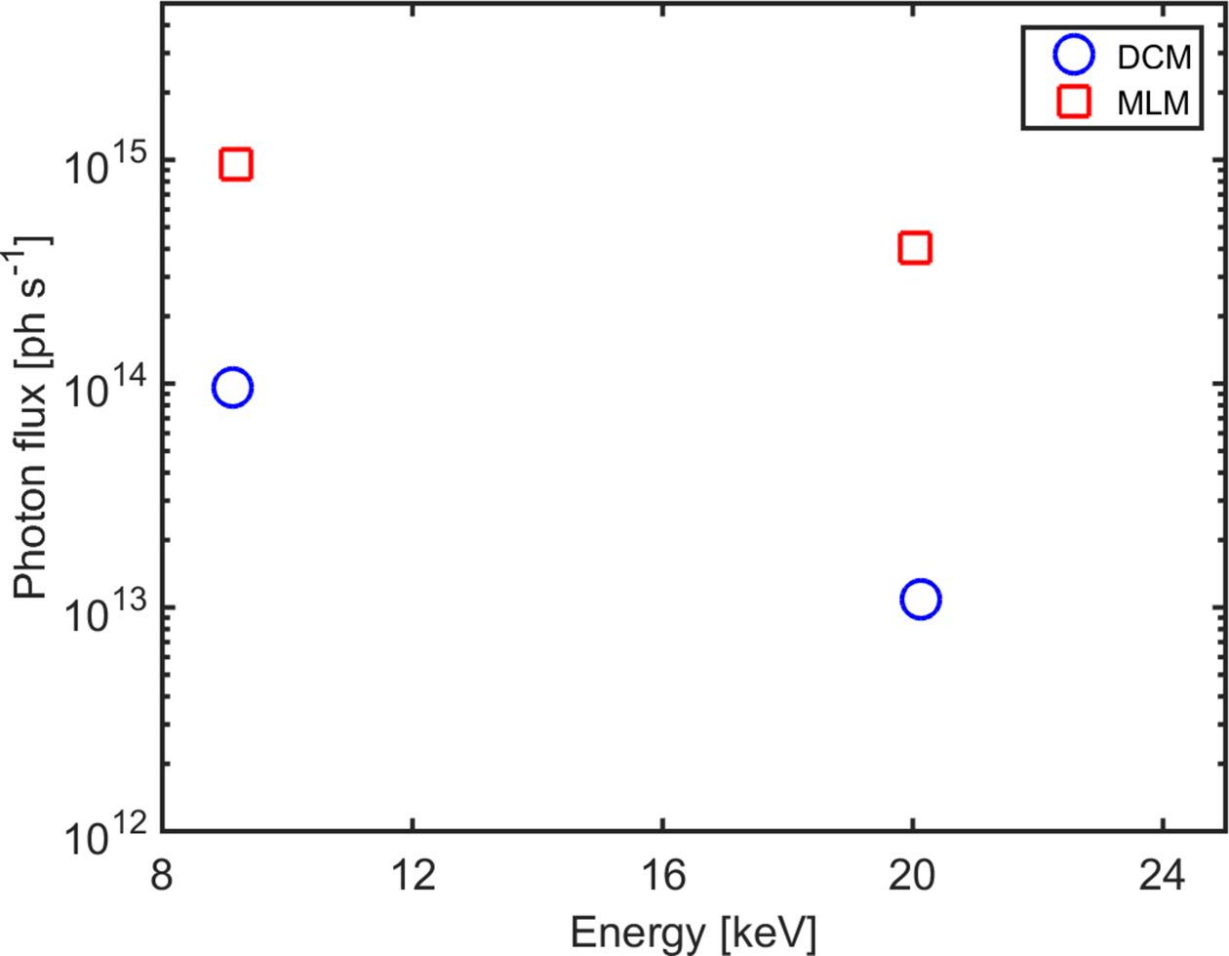
Measured X-ray photon flux at the sample position versus X-ray energy, shown for both monochromators and selected X-ray energies. The data were obtained using the minimum undulator gap and the maximum 24 µrad × 36 µrad (*x* × *y*) acceptance angle, *i.e.* the typical configuration for photon-hungry SRµCT and scanning SWAXS experiments.

The imaging techniques available on ForMAX rely on a large incident photon flux. In Fig. 8 ▸ we present the measured X-ray photon flux at the sample position for both monochromators. We collected the data with the minimum undulator gap (4.5 mm) and maximum acceptance angle (24 µrad × 36 µrad), as typically employed for photon-hungry SRµCT and scanning SWAXS experiments. We measured the flux of a strongly attenuated X-ray beam at ∼9 and 20 keV using the photon-counting EIGER detector, an approach that yielded reliable results owing to the efficient harmonic rejection using the Si and Rh stripes of the mirrors at these energies. The measured fluxes agree to within a factor of three with results based on ray-tracing simulations with *XRT* (Klementiev & Chernikov, 2014 ▸), assuming ideal undulator and optics.

Let us briefly discuss the available X-ray flux on ForMAX compared with competitive beamlines at third-generation synchrotron sources. In terms of SWAXS, the X-ray flux on the latter for typical experimental conditions in the energy range of ForMAX is generally ∼10^13^ photons s^−1^ or less [see *e.g.* Smith *et al.* (2021 ▸)]. The smaller X-ray beam divergence on ForMAX allows for a photon flux (using the DCM) of up to an order of magnitude larger than these values, greatly facilitating scanning SWAXS experiments. The MLM is available for niche experiments requiring an even higher flux. In terms of SRµCT, in turn, the flux on ForMAX (using the MLM) is comparable with that available at third-generation sources (Stampanoni *et al.*, 2006 ▸; Rau *et al.*, 2011 ▸; Vaughan *et al.*, 2020 ▸), albeit in an up to two orders of magnitude smaller beam cross section. We note that while the small beam size on ForMAX limits the capability of full-field imaging of large samples, as alluded to above, the very high photon density instead carries the potential for ultrafast imaging.

### 4.3. Coherence estimation

In this section, we present an initial quantification of the coherent properties on the ForMAX beamline. Specifically, we evaluate the effects of coherence in the formation of holographic fringes in an in-line holography experiment. We envisage performing an exhaustive analysis of the coherent properties of ForMAX (Goodman, 1985 ▸; Vartanyants & Singer, 2010 ▸) for different energies and imaging configurations, but this is out of the scope of the present paper.

**Figure 9:**
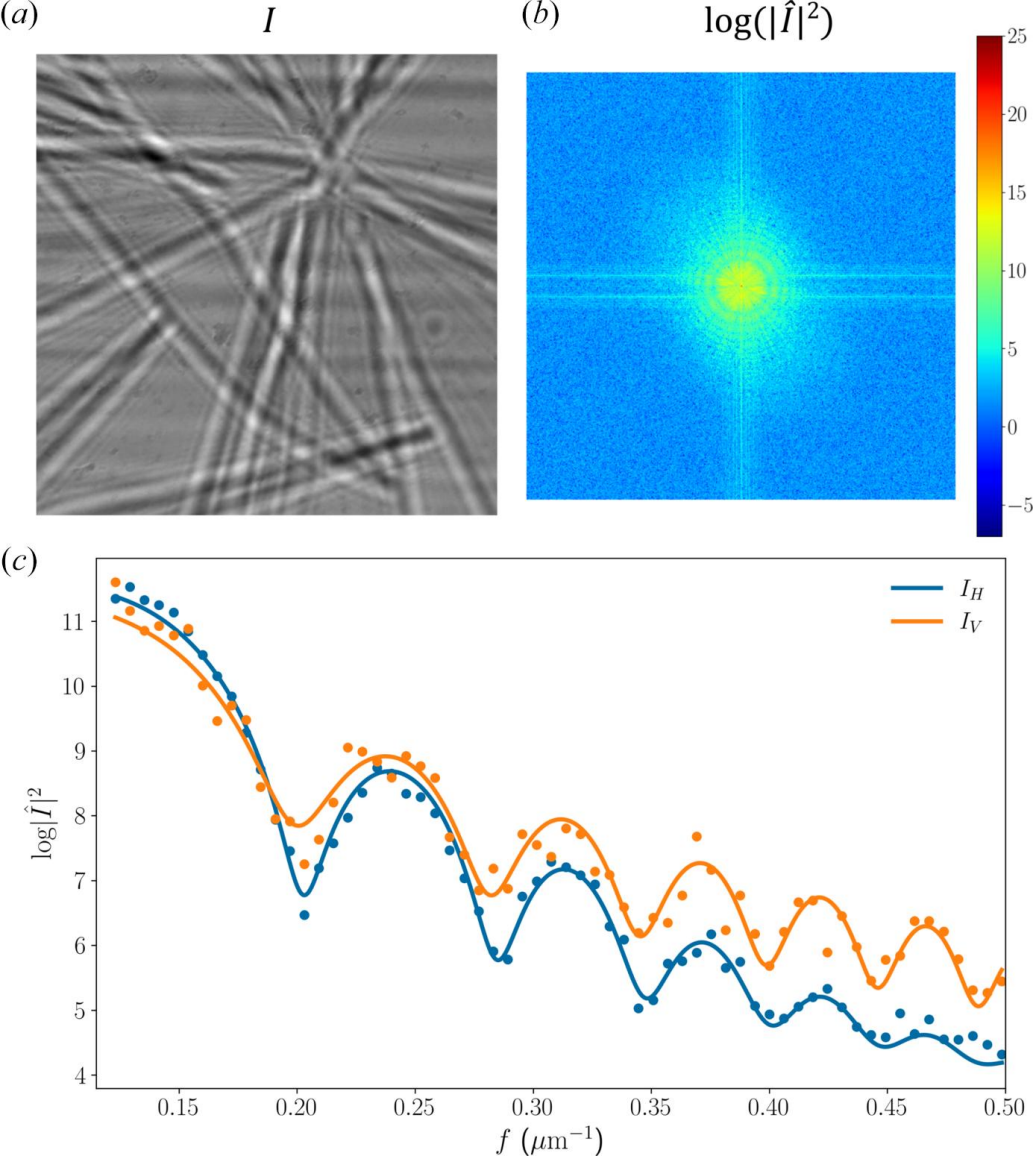
Coherence evaluation via contrast-transfer function analysis (CTF). (*a*) A hologram recorded from a broken Si_3_N_4_ membrane. (*b*) The power spectrum of the hologram in logarithmic scale ( 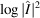 ). (*c*) Lateral (blue symbols) and vertical (orange symbols) components of the power spectrum. The solid lines depict fits to the data.

We performed in-line holography at 9.1 keV, imaging a broken Si_3_N_4_ membrane that exhibited several sharp edges with random orientations (Dierks & Wallentin, 2020 ▸). Fig. 9 ▸(*a*) depicts the hologram (*I*) recorded 19 cm downstream of the sample, using the SRµCT detection system with an effective pixel size of 0.325 µm and a response function (also known as the point spread function, PSF) with an FWHM corresponding to 3 pixels (σ_PSF_ = 0.41 µm), as estimated in Section 3.1 for the 20× magnification objective. The Fourier transform of the recorded hologram ( 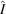 ) can be written as (Zabler *et al.*, 2005 ▸) 

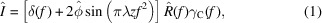

where *f* is the frequency, ϕ the wave’s phase after the object, λ the wavelength, *z* the propagation distance between the sample and the detector, *R* the detector’s point-spread function, and γ_C_ the degree of coherence. The sinusoidal term in equation (1) is also known as the contrast-transfer function (Guigay, 1977 ▸), and the visibility of its oscillations as a function of frequency is limited by the coherence and the PSF. The power spectrum ( 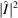 ) of the recorded hologram in logarithmic scale is depicted in Fig. 9 ▸(*b*). We clearly observe an asymmetry between the visibility of the CTF oscillations in the vertical and lateral directions due to coherence effects.

For an initial quantification of the coherence effects, we performed a fit of equation (1) to the power spectrum, describing the PSF and the degree of coherence by a Gaussian function with the standard deviation 

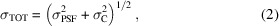

where σ_C_ is due to the degree of coherence. Because of the difference in phase space of the source in the principal directions, we fitted the data independently in the vertical and lateral directions as presented in Fig. 9 ▸(*c*). On the one hand, the vertical σ_TOT_ ≃ 0.40 µm is comparable to σ_PSF_, implying that the blurring of the fringes in the vertical direction is dominated by the PSF. This observation is compatible with the small vertical electron source size, and hence the large vertical coherence length, of Table 1 ▸. On the other hand, the lateral σ_TOT_ ≃ 0.71 µm corresponds to σ_C_ ≃ 0.58 µm, suggesting that the lateral visibility is mainly limited by the degree of coherence. This finding is in line with the larger lateral electron source size, and hence smaller lateral coherence length, of Table 1 ▸.

## 5. Probing hierarchical materials

The objective of the ForMAX beamline is to provide multiscale and multimodal structural characterization of materials from nanometre to millimetre length scales. In the following, we demonstrate this capability with a few examples.

### 5.1. Combined scanning SWAXS and SRµCT

**Figure 10:**
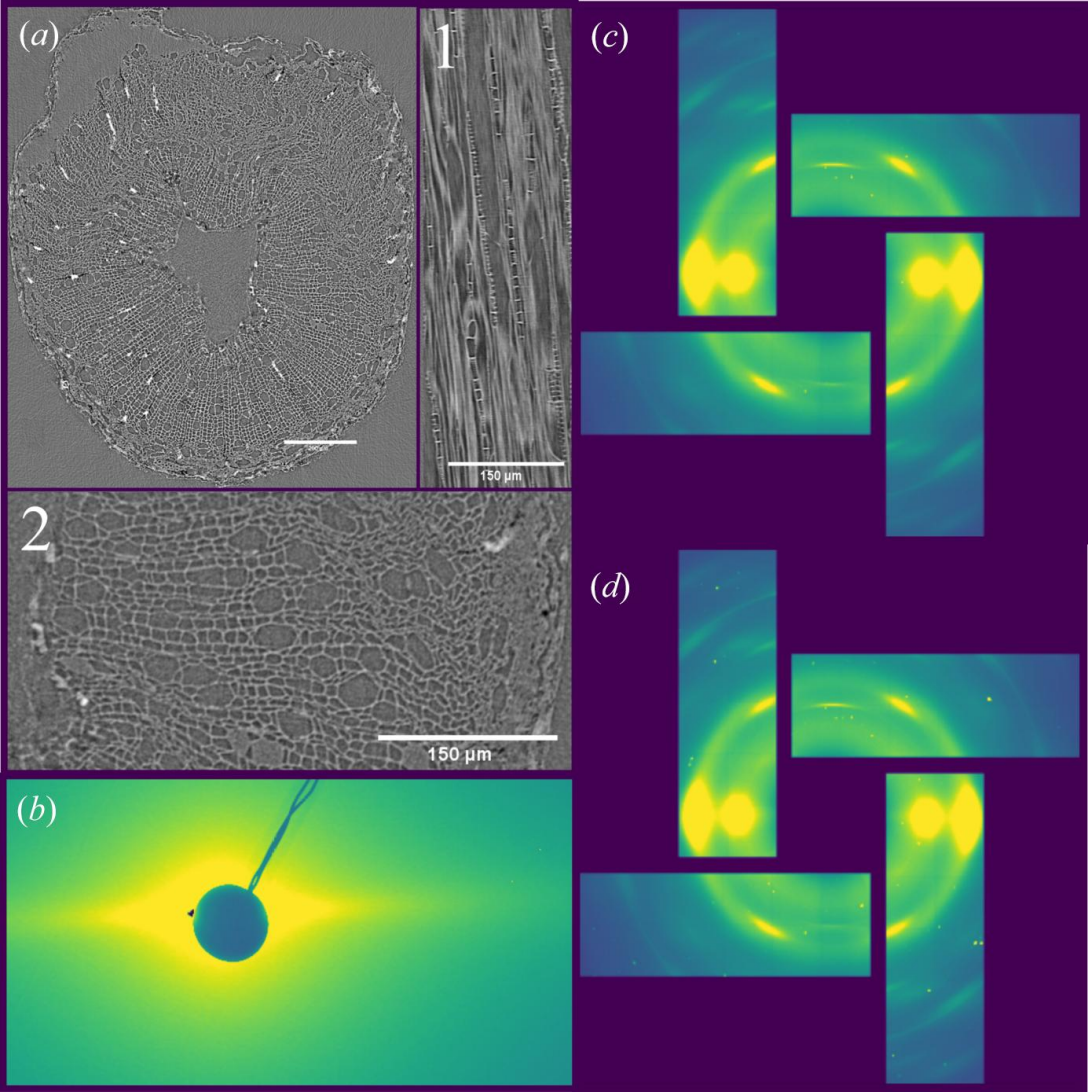
Zooming into hierarchical materials on ForMAX. Panel (*a*) shows a 2D slice from the reconstructed 3D volume of an aspen sapling with a magnified view into cellular structure in both tangential (*a*1) and radial directions (*a*2) as obtained by SRµCT. Such data provide microscopic structural characterization and allow users to identify regions of interest for nanoscale mapping. Panels (*b*) and (*c*)–(*d*) present local SAXS and WAXS data, respectively, acquired using an X-ray beam focused to ∼25 µm × 25 µm at the sample position. Panels (*c*) and (*d*) illustrate spatially resolved WAXS mapping of crystallites measured at different positions within the sample.

A key feature of ForMAX is the possibility of zooming into hierarchical materials, as illustrated in Fig. 10 ▸. In the first instance, we acquire a high-resolution 3D image by SRµCT, yielding microscopic structural characterization of the sample. This is exemplified in Fig. 10 ▸(*a*) for a sample of aspen sapling mounted in tangential geometry. The tomogram allows us to identify regions of interest (RoIs) for scanning SWAXS mapping of nanoscale structures. In the second instance, we focus the X-ray beam onto the sample position and collect spatially resolved SWAXS data on either the RoIs or the full sample, as illustrated in Figs. 10 ▸(*b*) and 10 ▸(*c*)–10 ▸(*d*) for SAXS and WAXS, respectively. Here, the anisotropy in the SAXS data is due to scattering from cellulose fibrils, whereas the main WAXS signal is due to diffraction from their crystalline parts. We note that whereas the SAXS and WAXS data of Fig. 10 ▸ provide access to structural properties such as microfibril size and orientation, the WAXS data of Figs. 10 ▸(*c*) and 10 ▸(*d*) also allow the mapping of other crystalline compounds within the sample, in this case calcium oxalate crystals. For bio-based materials, different crystalline agents are often present in the samples, and with the combination of spatial SRµCT and SWAXS data, the RoIs within the sample can be reconstructed using various scattering contrasts.

The feature of zooming into hierarchical materials is still under development. Potential means of improving user friendliness include, for example, a graphical user interface for selecting RoIs from the 3D SRµCT data. Nevertheless, ForMAX already provides in its present state a unique means of multiscale and multimodal structural characterization of soft and/or bio-based materials in the nanometre to millimetre range.

### 5.2. SAXS tensor tomography

As noted in the *Introduction* [Sec sec1], scanning SWAXS imaging provides structural characterization across seven orders of magnitude in length scales in a single experiment. SAXS tensor tomography (SASTT) is particularly useful for hierarchical materials, since the statistically averaged local orientation of fibrils, fibers or filaments accessible in these experiments is directly linked to the mechanical properties of the sample. In the scope of commissioning the beamline, we acquired a SASTT dataset from carbon fiber bundles that were carefully arranged in the shape of a small knot. A similar test sample has already been used in the initial SASTT commissioning experiments on the cSAXS beamline, Swiss Light Source (Liebi *et al.*, 2015 ▸). The purpose of such a measurement is to ensure the proper mapping of 3D reciprocal-space (scattering directions) and real-space directions, and *xy* scanning at different rotation and tilt orientations, into the reconstruction algorithm. We successfully reconstructed the first dataset already during the beam time, due to the readily available computing resources at the MAX IV high-performance cluster (HPC).

**Figure 11:**
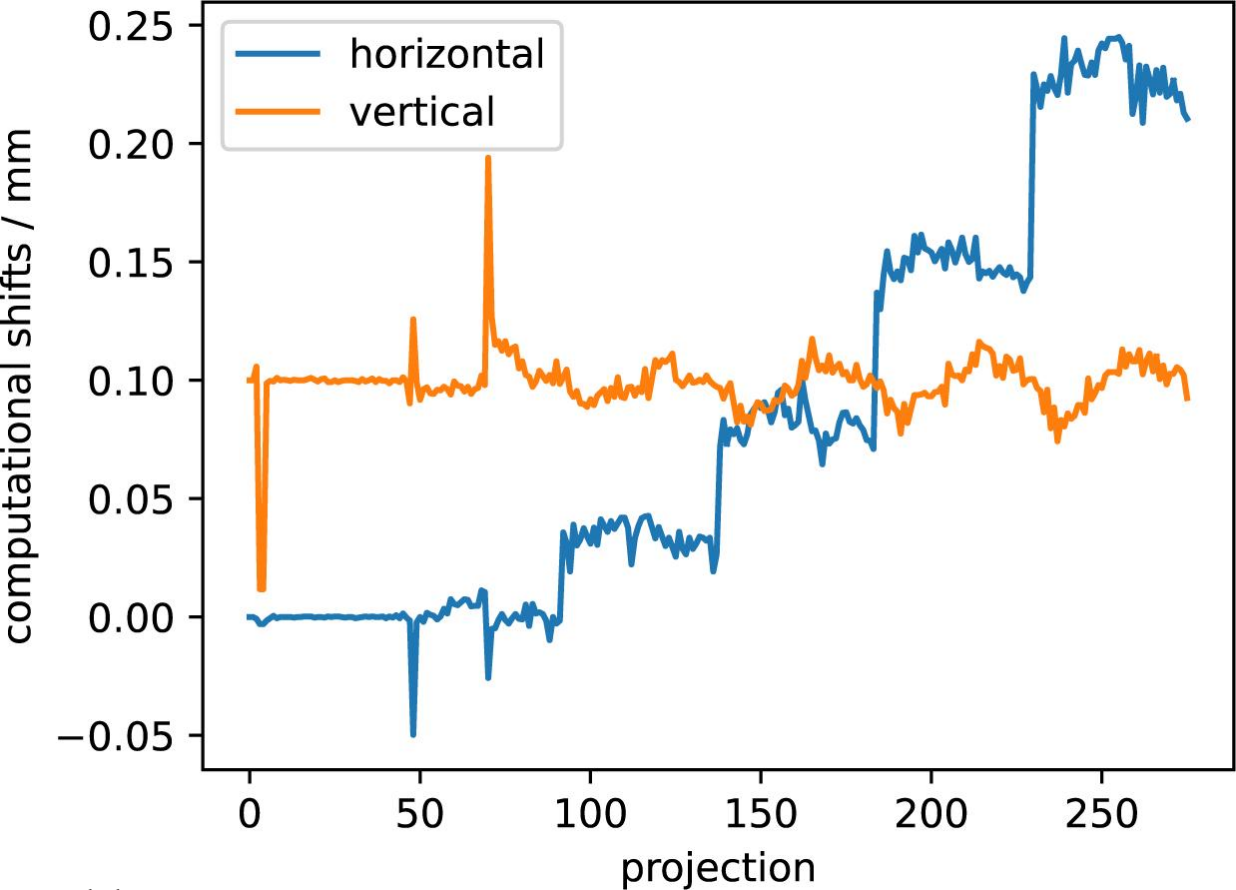
Horizontal and vertical shifts as computed by the alignment procedure and applied to all projections before being used as an input for the SASTT reconstructions via *Mumott*. Shifts are computed with the phase_cross_correlation function from the skimage.registration package in Python with a filtered back-projection (FBP) tomogram from the measurement at 0° tilt as a reference.

The input for the reconstruction consists of a dataset with 276 two-dimensional projections with 55 × 76 pixels (*x* × *y*) at a pixel size of 25 µm, computed from a total of 1.15 million detector frames. Each pixel of every projection consists of detector data which were reintegrated into 32 azimuthal bins in the range of *q* = 0.3–0.5 nm^−1^ and further symmetrically averaged to remove detector gaps. The remaining 16 azimuthal bins are used as input for the SASTT reconstruction with *Mumott* (Version 1.2; https://zenodo.org/records/8404162). Another important step in the workflow is projection alignment. We used a computational procedure to align all projections for different orientations of the sample (*Rx* = 0–180° for *Ry* = 0°, *Rx* = 0–360° for *Ry* > 0°) that first generates a tomogram for *Ry* = 0° and next back projects the projections of all other tilts and uses phase_cross_correlation from the skimage.registration Python package for image registration and computation of the required shifts. We used the integrated dark-field signal as input for the alignment procedure, due to the weak absorption signal from the carbon fibers. The same procedure was further used to mask out the sample holder and frame from some of the projections. The computed vertical and horizontal shifts are in total ≤300 µm (see Fig. 11 ▸), showing that the experimental setup is very stable.

We reconstruct the 3D reciprocal space in each voxel using band-limited Friedel symmetric spherical functions expressed in spherical harmonics up to a maximum order of 6, which results in 28 coefficients for each voxel that are then used to reconstruct the 3D reciprocal space. The orientation of the main structure is determined from the eigenvector associated with the smallest eigenvalue of the rank-2 tensor. We have checked the robustness of the reconstruction by visual comparison of 2D orientation, anisotropy and degree of orientation between the measurements and simulated projections of the reconstructed data. Finally, we calculate the degree of orientation as the ratio between the mean (isotropic component) and standard deviation (r.m.s. of anisotropic component).

**Figure 12:**
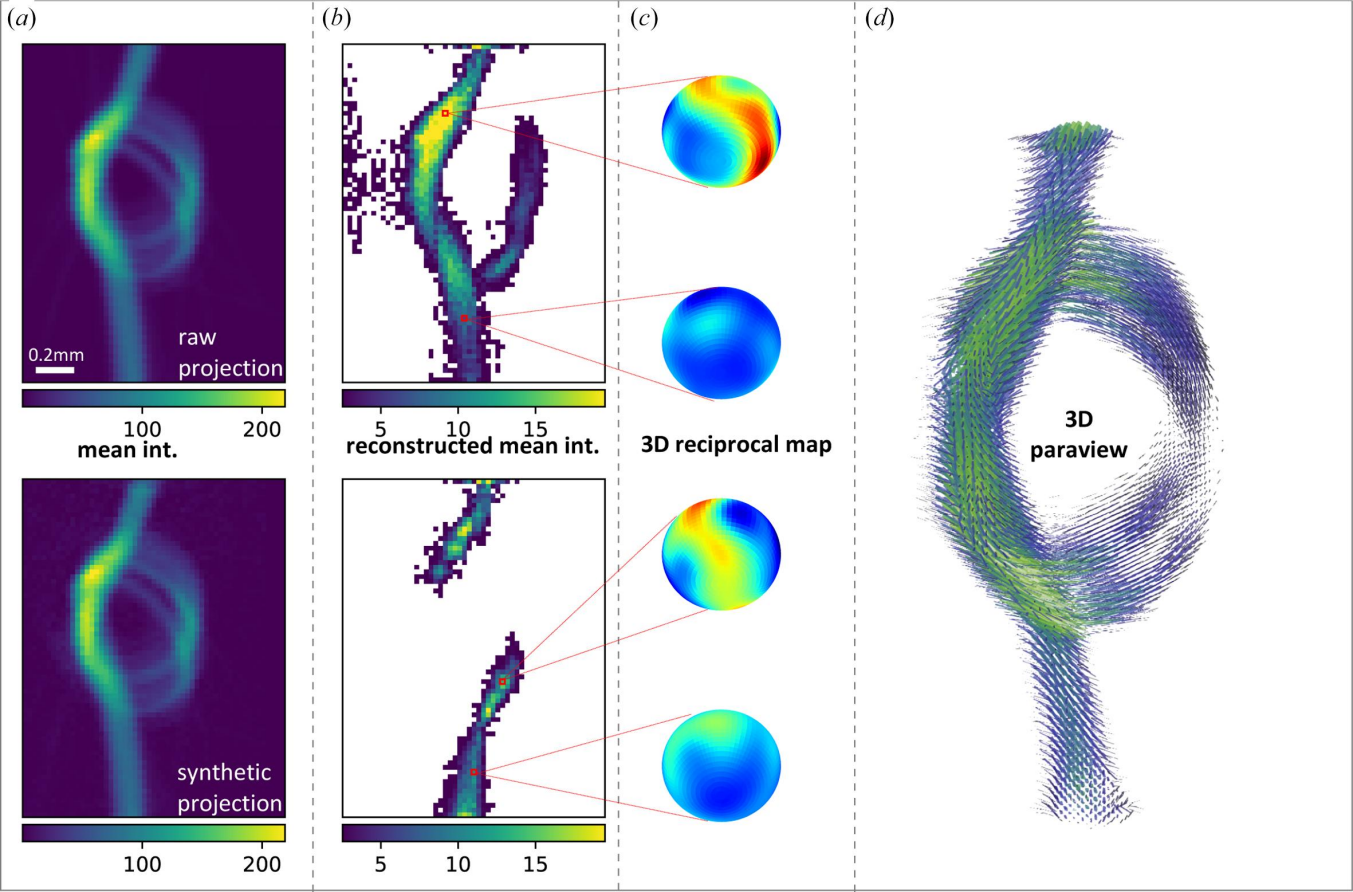
Summary of SASTT analysis of a carbon fiber knot. Part (*a*) compares the mean intensity of a measured projection with the corresponding synthetic projection computed from the reconstruction. In (*b*), two central cuts through the tomogram of the mean intensity (*zx* and *zy* slice) visualize the content of fibers throughout the tomogram. Four selected voxels are highlighted in red, for which the reciprocal-space maps are shown in (*c*) (interpolated with a 5° resolution and projected onto spheres). Panel (*d*) presents a *ParaView* rendering of the knot.

We display the results of the reconstruction in Fig. 12 ▸. In Fig. 12 ▸(*a*), we directly compare the input data of the mean intensity with a synthetic projection computed from the results of the reconstruction. Since there is essentially no difference between the measured and synthetic projections, which is the goal of the reconstruction, we move on to inspect the tomogram in more detail. Fig. 12 ▸(*b*) displays two central cuts, a *zx* and a *zy* slice through the tomographic reconstructed mean intensity, which give direct insights into the arrangement of the fibers within the knot. The top left of the image exhibits a region of higher intensity where the fiber bundles from top and bottom overlap, while the opposing side of the image shows two open loops of less densely packed material. Besides the mean intensity, SASTT reconstructions also offer the unique possibility of assessing the 3D reciprocal-space map within each voxel, as shown in Fig. 12 ▸(*c*) for selected voxels of the tomogram (scaled with the same color map for better comparison). The high-intensity region clearly shows a ring-like reciprocal-space map, which is expected for fiber-like structures. Finally, in Fig. 12 ▸(*d*) we visualize the combined information of the carbon fiber knot using the visualization software *ParaView* (https://www.paraview.org/). Cylinder glyphs with fixed aspect ratio point in the direction of the carbon fibers. We use the mean intensity, a measure of the material’s density, both to scale and to color-code the glyphs. Note that we have masked the output with a 3D array taken from the mean intensity to exclude low scattering regions and mask out data from air/background voxels.

### 5.3. Advanced rheological and mechanical testing

*In situ* rheological or mechanical testing is a common approach used to address, for example, flow-induced assembly of nanoparticles into advanced materials, or the relationship between structural and mechanical properties in fibrous materials. We foresee that such studies will be popular among our user community. However, while combined rheological and small-angle scattering experiments are rather mature (Eberle & Porcar, 2012 ▸), a deep understanding of the flow-induced assembly of nanoparticle suspensions into novel hierarchical materials requires simultaneous rheological and multiscale structural characterization (Kádár *et al.*, 2021 ▸, 2023 ▸). This is particularly important for the assembly and development of new materials from biomass, for which the importance of flow cannot be overstated. Likewise, while *in situ* uniaxial tensile or compressional load is commonly exerted during SWAXS or SRµCT experiments, materials engineering applications may require more complex load geometries and loading profiles or extensive load cycling under well controlled temperature and relative humidity. Again, this is of great importance for bio-based materials that are viscoelastic even in their solid state.

**Figure 13:**
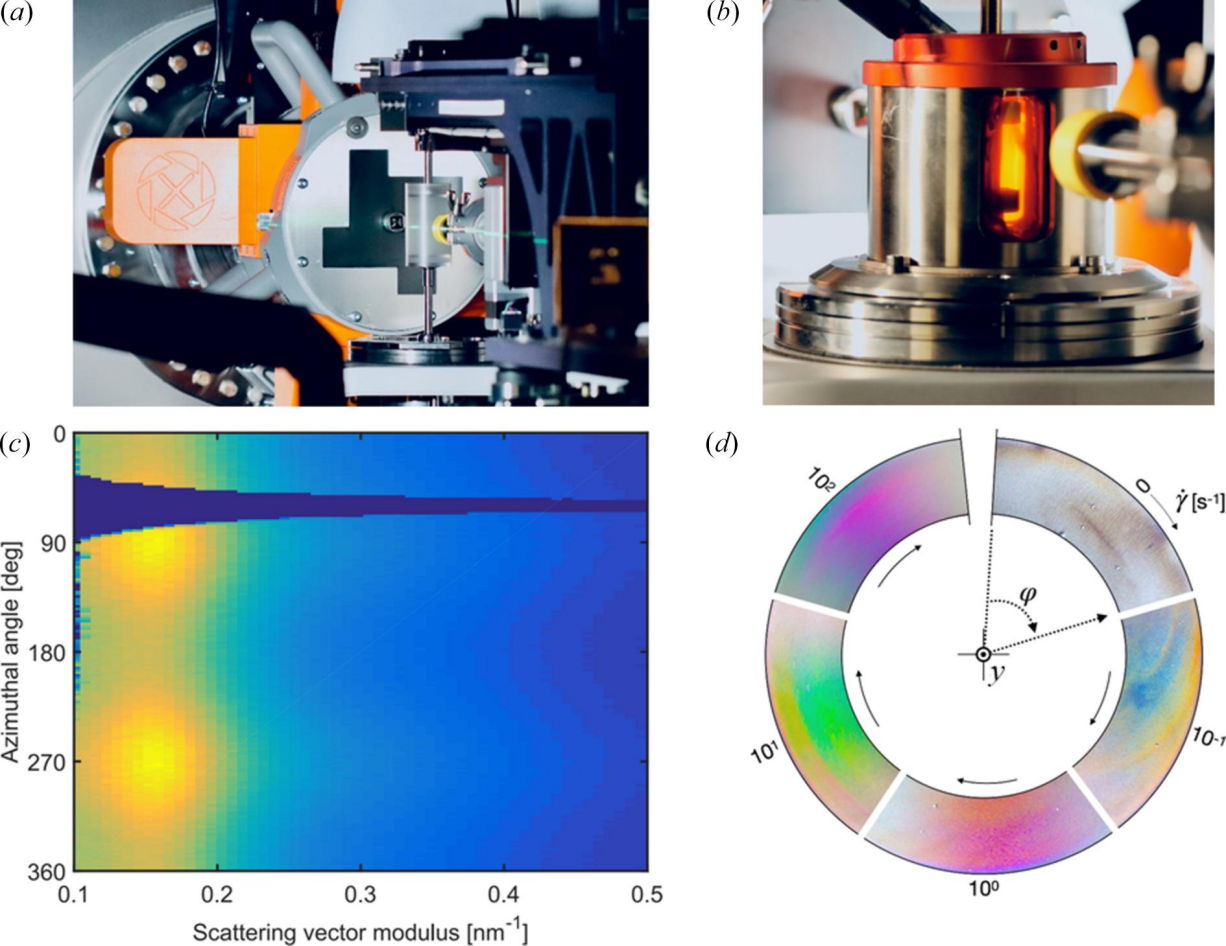
Examples of *in situ* rheological and mechanical testing on ForMAX. Panels (*a*) and (*b*) show the Rheo–SWAXS and DMA–SWAXS setups on ForMAX, respectively, based on the Anton Paar MCR702 rheometer available on the beamline. The SAXS data of panel (*c*) collected from a suspension of cellulose nanocrystals (CNCs), presented as a function of scattering vector modulus *q* and azimuthal angle φ, show nanoscale alignment of the suspension along the flow direction. Panel (*d*) illustrates *in situ* polarized light imaging of shear-induced mesoscale alignment in the *xz* plane of a CNC suspension as a function of shear rate 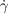 , with the same flow geometry sector visualized clockwise with increasing 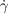 . The colored patterns contain information about mesoscale ordering of the CNC suspension.

In parallel with the construction and commissioning of ForMAX, we have therefore also developed an X-ray method­ology to expand further the possibilities for multiscale and multimodal structural characterization during rheological and mechanical testing. Based on this development work we can, together with our sister beamline CoSAXS (Kahnt *et al.*, 2021 ▸), provide users with the following capabilities:

(i) Simultaneous rheological and SWAXS experiments, as exemplified in Figs. 13 ▸(*a*) and 13 ▸(*c*) for a cellulose nanocrystal suspension subjected to laminar Couette flow in a concentric polycarbonate cup–bob geometry. Other geometries, including a plate–plate geometry that allows simultaneous mesoscale structural characterization by polarized light imaging [see Fig. 13 ▸(*d*)], and environmental control are also available. Finally, we note that the MLM provides the prospect of supreme temporal resolution in such studies.

(ii) We are addressing the need for more complex *in situ* load experiments by developing combined dynamic mechanical analysis (DMA) and SWAXS in an atmosphere of controlled temperature and humidity [Fig. 13 ▸(*b*)]. Inspired by recent development of combined rheological and SRµCT experiments (Dobson *et al.*, 2020 ▸), we are currently expanding the DMA–SWAXS experiments towards multiscale structural characterization by introducing simultaneous SRµCT capability, using the rheometer in co-rotation mode as the tomographic rotation stage.

## 6. Conclusions and outlook

We have recently brought into operation the beamline ForMAX that allows unique multiscale and multimodal structural characterization of hierarchical materials in the nanometre to millimetre range by combining SWAXS, scanning SWAXS imaging and SRµCT (or any combination of these techniques) in a single experiment. Although we are still optimizing the beamline’s performance, the initial benchmarking of the X-ray beam properties reported here demonstrates ForMAX’s potential.

A major aspect of ForMAX is the possibility of monitoring multiscale structural evolution during material processing. Currently we are developing this possibility along two different paths. First, the very high photon density on ForMAX provides unprecedented possibilities for ultrafast full-field imaging. Second, we are working on dedicated sample environments that allow multiscale structural characterization during complex rheological or mechanical testing under controlled temperature and humidity, as exemplified above. We hope to make these developments available for general users in the near future.

## Figures and Tables

**Table 1:** Main parameters of the MAX IV 3 GeV storage ring

Storage ring energy	3 GeV
Circumference	528 m
Beam current (operation November 2023)	400 mA
Projected beam current	500 mA
Electron beam emittance (*x* × *y*)	326 pm rad × 8 pm rad
Electron energy spread	7.7 × 10^−4^
Electron source size (*x* × *y*)	54 µm × 4 µm
Electron source divergence (*x* × *y*)	6 µrad × 2 µrad
Top up	Every 10 minutes

**Table 2:** Main components of the beamline

Component	Distance from source (m)
Undulator	0
Front-end movable mask	19.5
White-beam slits	23.9
Double-multilayer monochromator	25.0
Double-crystal monochromator	27.0
Vertically focusing mirror	30.2
Horizontally focusing mirror	31.0
Monochromatic slits	28.1, 32.3, 36.3, 41.5–41.8
X-ray prism lens (placeholder)	36.6
Compound refractive lenses	40.5
Experimental table	42.0
Full-field microscope	42.0–42.3
WAXS detector	42.1
SAXS detector	42.8–49.6

**Table 3:** Main parameters of the undulator

Magnet material	NdFeB
Pole material	Vanadium Permedur
Energy range	8–25 keV
Period length	17 mm
Number of periods	166
Minimum magnetic gap	4.5 mm
*K* value at minimum gap	1.89
Phase error	≤2.5°
Total power†	∼11.5 kW

† At projected 500 mA ring current.

**Table 4:** Hybrid photon-counting pixel detectors available on the beamline

	Dectris	X-Spectrum
	EIGER2 X 4M	Lambda 3M
Number of pixels	4 million	3 million
Sensor size	2068 × 2162 pixels	4 × 516 × 1536 pixels
Pixel size	75 µm × 75 µm	55 µm × 55 µm
Sensor material	Si	Si
Sensor thickness	450 µm	320 µm
Dynamic range	32 bit	24 bit
Maximum frame rate†	560 Hz	1000 Hz
Data storage	Streaming	Streaming
Speciality	Vacuum compatible	‘Windmill’ shaped

† Full dynamic range.

**Table 5:** sCMOS cameras available on the beamline

	Hamamatsu	Andor	Photron
	ORCA Lightning	Zyla 5.5	FASTCAM Nova S16
No. of pixels	12 million	5.5 million	1 million
Sensor size	4608 × 2592 pixels	2560 × 2160 pixels	1024 × 1024 pixels
Pixel size	5.5 µm × 5.5 µm	6.5 µm × 6.5 µm	20 µm × 20 µm
Maximum dynamic range	16 bit	16 bit	12 bit
Maximum frame rate†	121 Hz (12 bit)	100 Hz (12 bit)	16 kHz (12 bit)
	30 Hz (16 bit)	75 Hz (16 bit)	
Data storage	Streaming	Streaming	128 GB / 4 TB

† Full frame.

## Footnotes

1 Throughout this paper, we define the resolution of mechanical components as the minimum incremental motion per full step.

2 Depending on the setup, the SAXS detector may be partially shadowed at short sample-to-detector distances.

3 In the case of the high-speed Photron sCMOS camera, the data will temporarily be saved on the local data storage of the camera, before transfer to the central data storage.
